# Genome-wide interaction and pathway-based identification of key regulators in multiple myeloma

**DOI:** 10.1038/s42003-019-0329-2

**Published:** 2019-03-04

**Authors:** Subhayan Chattopadhyay, Hauke Thomsen, Pankaj Yadav, Miguel Inacio da Silva Filho, Niels Weinhold, Markus M. Nöthen, Per Hoffman, Uta Bertsch, Stefanie Huhn, Gareth J. Morgan, Hartmut Goldschmidt, Richard Houlston, Kari Hemminki, Asta Försti

**Affiliations:** 1 Division of Molecular Genetic Epidemiology, German Cancer Research Center (DKFZ), Heidelberg, 69120 Germany; 2 Faculty of Medicine, University of Heidelberg, Heidelberg, 69117 Germany; 3 University Clinic Heidelberg, Internal Medicine V, Heidelberg, 69117 Germany; 4 Myeloma Institute, University of Arkansas for Medical Sciences, Little Rock, 72205 AR USA; 5 Institute of Human Genetics, University of Bonn, Bonn, 53127 Germany; 6 Department of Genomics, Life & Brain Research Center, University of Bonn, Bonn, 53127 Germany; 7 Department of Biomedicine, University of Basel, Basel, 4003 Switzerland; 8 National Centre of Tumor Diseases, Heidelberg, 69120 Germany; 9 Division of Genetics and Epidemiology, The Institute of Cancer Research, London, SW7 3RP UK; 10 Division of Molecular Pathology, The Institute of Cancer Research, London, SW7 3RP UK; 11 Center for Primary Health Care Research, Lund University, 205 02 Malmö, Sweden

## Abstract

Inherited genetic susceptibility to multiple myeloma has been investigated in a number of studies. Although 23 individual risk loci have been identified, much of the genetic heritability remains unknown. Here we carried out genome-wide interaction analyses on two European cohorts accounting for 3,999 cases and 7,266 controls and characterized genetic susceptibility to multiple myeloma with subsequent meta-analysis that discovered 16 unique interacting loci. These risk loci along with previously known variants explain 17% of the heritability in liability scale. The genes associated with the interacting loci were found to be enriched in transforming growth factor beta signaling and circadian rhythm regulation pathways suggesting immunoglobulin trait modulation, T_H_17 cell differentiation and bone morphogenesis as mechanistic links between the predisposition markers and intrinsic multiple myeloma biology. Further tissue/cell-type enrichment analysis associated the discovered genes with hemic-immune system tissue types and immune-related cell types indicating overall involvement in immune response.

## Abstract (web summary)

Subhayan Chattopadhyay et al. conduct genome-wide interaction studies to identify genetic susceptibility to multiple myeloma. They find 16 unique interacting loci, which implicate immune response in multiple myeloma pathology.

## Introduction

Multiple myeloma is the second most prevalent hematological malignancy with almost 31,000 estimated new diagnoses in the United States in 2018^1^. Multiple myeloma, a B-cell neoplasm, is characterized by proliferation of clonal plasma cells in bone marrow. Familial aggregation of multiple myeloma suggests predisposition due to inherited genetic variation^2,3^. Susceptibility to multiple myeloma and its genetic relationship with the related diseases, monoclonal gammopathy of unknown significance (MGUS), and amyloid light chain (AL) amyloidosis, have lately been established through genome-wide association studies (GWASs)^4–6^. Although a total of 23 risk loci have been discovered predisposing to multiple myeloma, they are estimated to explain only about 16% of the heritability^5,7^. Moreover, genetic heterogeneity among multiple myeloma tumors bears complication in characterization of genetic susceptibility to multiple myeloma and in understanding of clinical consequences^8,9^.

In addition to the linear association analysis, we have recently identified several inherited risk loci predisposing to MGUS through genome-wide genetic interaction^10^. To gain ample insight into genetic predisposition of multiple myeloma, we performed here the first genome-wide interaction study using two patient cohorts comprising a total of 3999 cases and 7266 controls. We extended the investigation with a subsequent meta-analysis of the two cohorts to increase the statistical power of detection. We also examined enrichment of expression of the identified genes in several tissue and cell types. Additionally, we performed gene set enrichment and pathway analyses to confer a biological understanding to our investigation. Collectively, our analyses support the hypothesis that genetic interaction plays a crucial role in multiple myeloma predisposition. The sentinel genes thus discovered are often expressed in tissues and cell lineages of hematopoietic system responsible for immune-modulation and they also influence inherited susceptibility to multiple myeloma through regulation of circadian rhythm and Smad-dependent TGFβ pathways.

## Results

### Interacting chromosomal loci

**Fig. 1:**
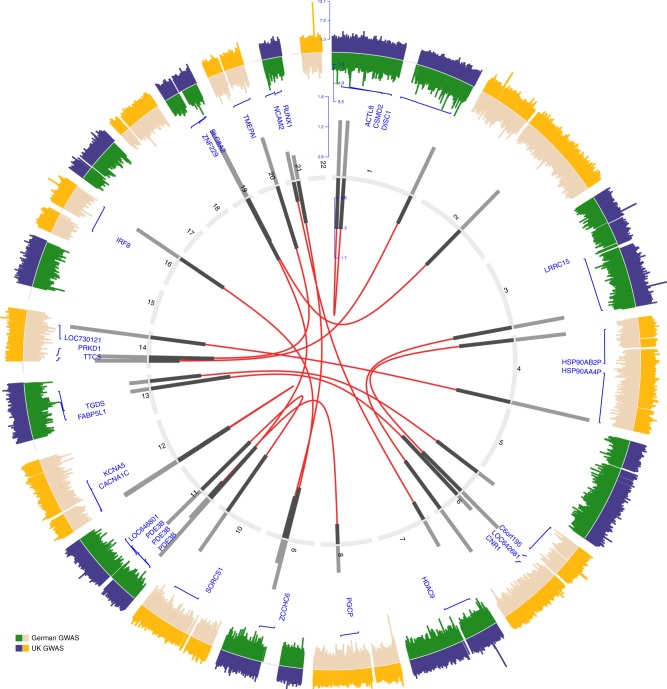
Interaction analysis identifies 16 unique risk loci pairs. Circos plot of genome-wide association and significant interaction results for the identified paired risk loci. The two outer most panels display results from genome-wide association study on a Manhattan plot for autosomal variants on a negative log transformed scale. Inner numbered panel represents the chromosomes and effect-sizes of significant interacting pairs are plotted on bar charts from both samples (dark: German sample; light: UK sample). Interacting pairs are line joined in the inner most panel based on their chromosomal positions (NCBI build 19 human genome). Annotations of single-nucleotide polymorphisms to gene ids are displayed on the inner manhattan plot

Two quality controlled sets of genotyped data consisting 2282 cases and 5197 controls from the UK and 1717 cases and 2069 controls from Germany were subjected to pairwise interaction analysis accounting for 0.43 million and 0.52 million single-nucleotide polymorphisms (SNPs), respectively. Meta-analysis of associative linear interaction on transformed correlation statistics rendered 16 unique SNP pairs belonging to 16 exclusive chromosomal regions reaching genome-wide threshold of 5.0 × 10^−10^ (Fig. 1 and Supplementary Data 1).

The strongest meta-analyzed signal was provided by an interaction between rs7048811 at 9q21.31 (associated gene *GNAQ*) and rs7204305 at 16q24.1 (*IRF8*) (OR_Meta *=* _1.22; 95% CI = 1.12–1.32; *P* = 1.3 × 10^–10^, Supplementary Data 1). This interaction was consistent in both cohorts with a conservative level of significance (UK cohort: OR = 1.20, 95% CI = 1.08–1.33, *P* = 7.0 × 10^–06^; German cohort: OR = 1.24, 95% CI = 1.09–1.41, *P* = 7.6 × 10^–06^). The highest statistically significant OR was observed for the second most strong interaction signal between rs2167453 at 11p15.2 (*PDE3B*) and rs2734459 at 19q13.31 (*ZNF229*) (OR_Meta *=* _1.52, 95% CI = 1.33–1.73, *P* = 1.3 × 10^–10^).

### Biological inference of the interacting chromosomal loci

Many of the risk SNPs identified, although showing promising genotypic interactions, are mapped to non-coding regions of the genome and possibly contribute to multiple myeloma etiology by affecting gene expression^11^. In order to gain biological understanding of the newly identified interacting risk loci, we interrogated expression quantitative trait locus  (eQTL) data generated on malignant plasma cells obtained from patients of the German multiple myeloma trials. Strongest eQTL signals were observed by rs2167453 at 11p15.2 for cytochrome P450, family 2, subfamily R, polypeptide 1 (*CYP2R1*) and by rs923934 at 3q29 for family with sequence similarity 43, member A (*FAM43A*), both with$P_{eQTL} = 4.40 \times 10^{ - 5}$ (Table 1). Also the interacting partners of these SNPs served as eQTLs with a moderate signal, rs2734459 for CLASRP, ZNF224, and APOE and rs13201167 for AKAP12 and C6orf211.

**Table 1:** Genome-wide association study (GWAS) summary-data-based Mendelian randomization (SMR)

Probe	Gene name	Gene ID	Single- nucleotide polymorphism (SNP) ID	eQTL *P*-value	GWAS *P*-value	SMR *P*-value
9364_at	RAB28, member RAS oncogene family	RAB28	rs17362130	1.14E-03	3.68E-05	4.84E-03
8737_at	Receptor (TNFRSF)-interacting serine-threonine kinase 1	RIP1	rs6918808	1.23E-03	4.01E-05	5.04E-03
7289_at	Tubby like protein 3	TULP3	rs2238087	1.14E-03	2.58E-04	1.27E-02
808_at	Calmodulin 3 (phosphorylase kinase, delta)	CALM3	rs4802363	1.76E-03	1.99E-03	1.28E-02
11133_at	Kaptin (actin binding protein)	KPTN	rs4802363	1.98E-03	2.91E-03	1.30E-02
8605_at	Phospholipase A2, group IVC (cytosolic, calcium-independent)	PLA2G4C	rs4802363	1.72E-03	4.62E-03	1.33E-02
57820_at	Cyclin B1 interacting protein 1, E3 ubiquitin protein ligase	CCNB1lP1	rs10130942	1.41E-03	3.98E-03	1.86E-02
10082_at	Glypican 6	GPC6	rs17181808	1.06E-03	6.41E-04	1.86E-02
1690_at	coagulation factor C homolog, cochlin (Limulus polyphemus)	COCH	rs12436395	3.52E-04	1.88E-02	2.03E-02
120227_at	Cytochrome P450, family 2, subfamily R, polypeptide 1	CYP2R1	rs2167453	4.40E-05	2.56E-02	2.10E-02
579_at	NK3 homeobox 2	NKX3–2	rs17362130	2.11E-03	1.18E-03	2.72E-02
80759_at	KH homology domain containing 1	KHDC1	rs4706511	1.01E-03	5.49E-03	3.47E-02
10553_at	HIV-1 Tat interactive protein 2, 30 kDa	HTATIP2	rs10766743	1.85E-03	2.11E-03	3.60E-02
79624_at	Chromosome 6 open reading frame 211	C6orf211	rs13201167	4.40E-04	2.47E-03	3.65E-02
160897_at	G protein-coupled receptor 180	GPR180	rs17181808	4.40E-04	5.04E-03	3.66E-02
5272_at	Serpin peptidase inhibitor, clade B (ovalbumin), member 9	SERPINB9	rs6918808	1.01E-03	3.04E-03	3.80E-02
23483_at	TDP-glucose 4,6-dehydratase	TGDS	rs17181808	4.84E-04	6.32E-03	3.82E-02
440145_at	Mitotic spindle organizing protein 1	MZT1	rs17089906	2.64E-04	9.85E-03	4.20E-02
9590_at	A kinase (PRKA) anchor protein 12	AKAP12	rs13201167	2.16E-03	3.63E-03	4.27E-02
688_at	Kruppel-like factor 5 (intestinal)	KLF5	rs17089906	1.76E-04	2.01E-02	4.54E-02
7767_at	Zinc finger protein 224	ZNF224	rs2734459	7.04E-04	3.59E-03	4.66E-02
348_at	Apolipoprotein E	APOE	rs2734459	1.23E-03	5.19E-03	5.55E-02
81029_at	Wingless-type MMTV integration site family, member 5B	WNT5B	rs2238087	1.98E-03	6.45E-03	5.65E-02
404550_at	Chromosome 16 open reading frame 74	C16orf74	rs7204305	1.98E-03	6.74E-03	5.67E-02
11129_at	CLK4-associating serine/arginine rich protein	CLASRP	rs2734459	2.64E-04	1.32E-02	8.43E-02
131583_at	Family with sequence similarity 43, member A	FAM43A	rs923934	4.40E-05	1.90E-02	8.89E-02

**Fig. 2:**
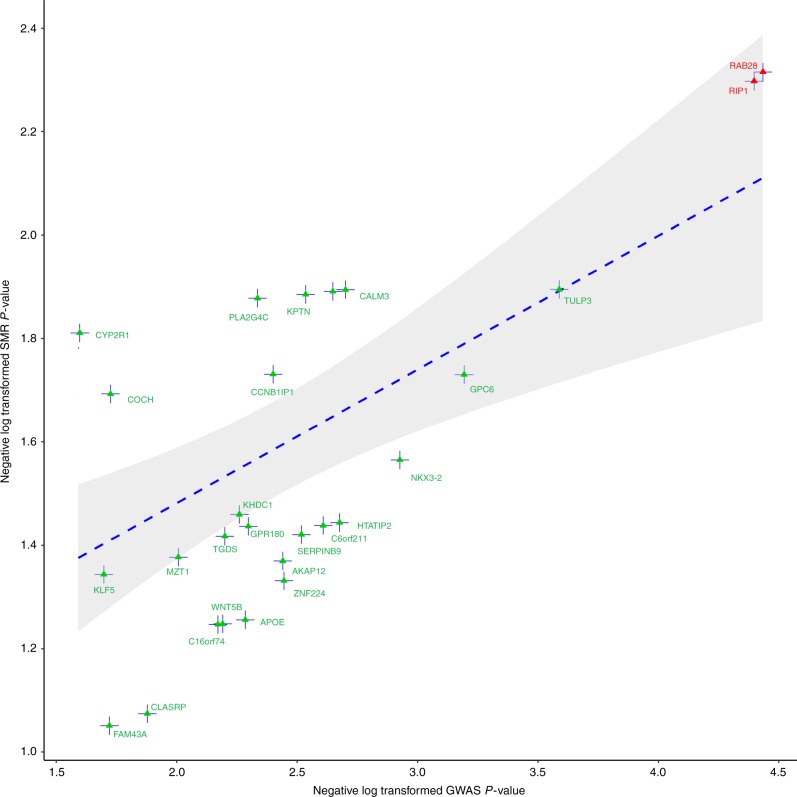
Summary-data-based Mendelian randomization analysis of interaction detected multiple myeloma risk loci and gene expression in plasma cells. Negative log transformed *P*-values are plotted from GWAS against that of SMR identified causal cis-eQTLs at suggestive level. Top two significant elements are annotated in red. The blue line represents fitted liner regression representing linear association and the shaded region encompasses 95% confidence interval

Summary-data-based Mendelian randomization (SMR) was employed to analyze pleiotropic effects between the GWAS signal and the cis-eQTL for genes residing within 1 Mb window of the interacting SNP loci to identify causal relationship between variants and disease phenotype via instrumentation of gene regulation^12^. The strongest pleiotropic signal was observed at 4p15.33 by rs17362130 for RAS oncogene family member 28, *RAB28* $\left( {P_{SMR} = 4.84 \times 10^{ - 3}} \right)$ and at 6p25.2 by rs6918808 for receptor (TNFRSF)-interacting serine/threonine kinase 1, *RIPK1* ($P_{SMR} = 5.04 \times 10^{ - 3}$, Table 1 and Fig. 2), respectively. Oncogenic ras family members are frequently mutated in multiple myeloma^13,14^. RIPK1 interacts with RIPK3 to activate the necrosome complex that is responsible for instigation of several death receptors, which can induce apoptosis, necroptosis, or cell proliferation^15^. rs17362130 is also an eQTL for *NKX3–2* with a moderate signal $\left( {P_{eQTL} = 2.11 \times 10^{ - 3}} \right)$ and rs6918808 for *SERPINB9*. NKX3–2 is involved in skeletal development^15^. SERPINB9 is a known inhibitor of granzyme and may mediate tumor immune evasion by apoptosis inhibition^16,17^.

**Fig. 3:**
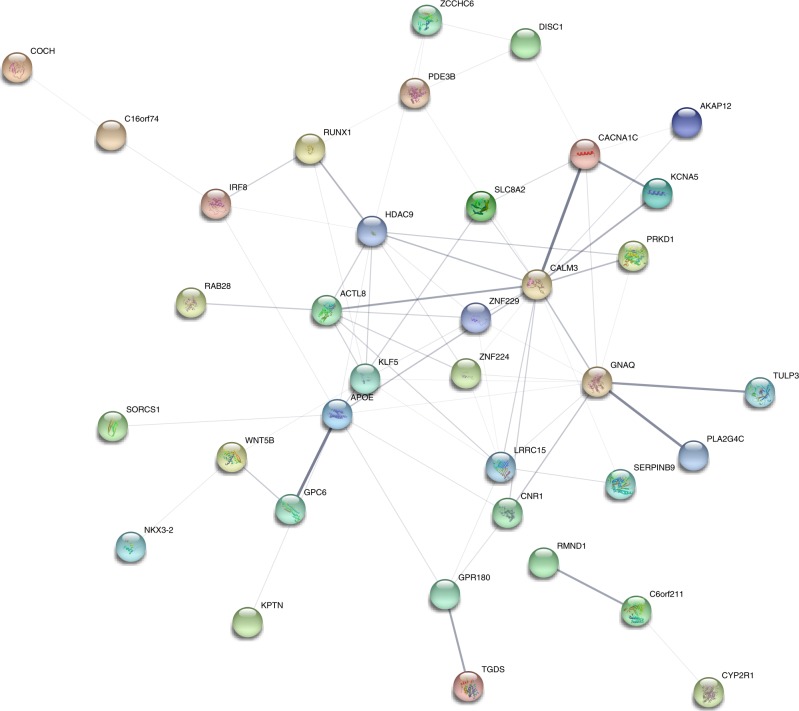
Genetic network enrichment plot. All nodes represent direct annotations of interaction-identified elements or first order interactions. Thickness of the edges represent enrichment robustness between connecting nodes based on emperical evidence gathered from curated database (cyan), experimentally determined (magenta), gene neighborhood (forest green), gene fusion (red), gene co-occurrence (navy blue), text mining (lawn green), co-expression (black), protein homology (lavender indigo). Node color signifies different/shared protein functionality. Additional nodes are considered based on prediction score ≥ 0.99

We investigated shared biological and information driven connections between the genes annotated to the variants by creating a genetic network. Unique annotations from the 16 interaction-identified variants along with the SMR identified causally related genes were subjected to network enrichment and a single batch of first order interacting genes based on data-mined enrichment index were additionally added to increase confidence of network associations (Fig. 3).

**Fig. 4:**
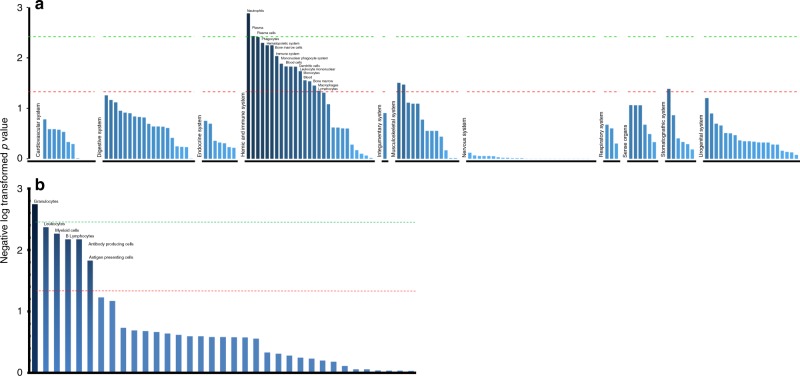
Tissue and cell type enrichment plots. **a** Tissue enrichment identifies significant tissue types mostly affected with interaction-identified genes. **b** Cell type enrichment analysis identifies cells with observed expression regulation of the same candidates

We applied Data-Driven Expression-Prioritized Integration for Complex Traits (DEPICT) for in silico analyses of enrichment of expression of genes annotated to the associated loci in tissues and cell types. To this end interaction-identified SNPs were clustered to 12 unique loci and were tested for significant excess expression of the corresponding genes in 209 Medical Subject Heading (MeSH) annotations against 37,427 microarrays procured in backend. Twenty-seven tissue or cell type annotations were discovered significant at a suggestive level (*P* < 0.05); 16 of them belonged to the hemic and immune system, two to the musculoskeletal system and one to the stomatognathic system (Fig. 4a), as well as six cell types related to hematopoietic system (Fig. 4b and Supplementary Data 2).

### Biological inference of the GWAS-identified loci

Next, we investigated functional relationships among the previous GWAS-identified loci using the pathway analysis tool PASCAL with the bottom-up approach. To avoid possible complications arising from statistical convergence of the test statistic, we used sum of chi-square statistic to test for functional association against pathways extracted from REACTOME, KEGG, and BIOCARTA libraries (Supplementary Data 3). A total of 12 enriched pathways reached a global threshold of 0.0025 for the combined *P*-value. Three of the pathways, thus, detected were signaling cascades reflecting the activation status of the SMAD family proteins, as signal transducers for receptors of the cytokine TGFβ represented by *SMAD2, SMAD3, SMAD4* heterotrimer regulates transcription, $P_{\mathrm{combined}} = 5.70 \times 10^{ - 3},$ TGFβ receptor signaling activates *SMADs*, $P_{\mathrm{combined}} = 8.60 \times 10^{ - 3}$ and transcriptional activity of *SMAD2, SMAD3, SMAD4* heterotrimer, $P_{\mathrm{combined}} = 1.49 \times 10^{ - 2}$. Additionally, two pathways related to the regulation of circadian rhythms mediated by two nuclear receptor proteins retinoic acid receptor-related orphan receptor A (*RORA*) and Rev-ErbA were identified: Circadian repression of expression by REV-ERBA, $P_{\mathrm{combined}} = 5.52 \times 10^{ - 4}$ (Table 2) and *RORA* activates circadian expression. $P_{\mathrm{combined}} = 2.13 \times 10^{ - 3}$. Also, modulation of *ALK* receptor tyrosine kinase activity was indicated with ALK pathway, $P_{\mathrm{combined}} = 2.82 \times 10^{ - 3}$.

**Table 2:** Pathway enrichment analysis with PASCAL detects 12 putative pathways related to multiple myeloma. Combined *P*-values are obtained with Brown’s method. *P*_*X*_ denotes *P*-value obtained from interaction test of set *X*

Database	Pathway	$P_{\mathrm{Ger}}$	$P_{\mathrm{UK}}$	$P_{\mathrm{Meta}}$	$P_{\mathrm{Combined}}$
REACTOME	Circadian repression of expression by REV-ERBA	3.50E-04	1.45E-01	4.16E-03	5.52E-04
	APOBEC3G mediated resistance to HIV infection	5.79E-02	1.74E-03	2.09E-03	1.02E-03
	RORA activates circadian expression	1.24E-03	1.83E-01	1.20E-02	2.13E-03
	Deposition of new CENP-A containing nucleosomes as the centromere	7.00E-02	7.49E-03	3.82E-03	4.48E-03
	SMAD2 SMAD3 SMAD4 heterotrimer regulates transcription	8.83E-02	7.81E-03	1.88E-02	5.70E-03
	TGFβ receptor signaling activates SMADs	1.73E-02	6.39E-02	4.38E-03	8.60E-03
	GABAA receptor activation	2.36E-02	6.27E-02	1.62E-02	1.11E-02
	Iron uptake and transport	4.84E-02	4.20E-02	8.91E-03	1.46E-02
	Transcriptional activity of SMAD2 SMAD3 SMAD4 heterotrimer	9.53E-02	2.18E-02	4.15E-02	1.49E-02
	Purine salvage	8.82E-02	2.51E-02	3.71E-02	1.57E-02
	Apoptosis induced DNA fragmentation	1.76E-02	1.29E-01	2.32E-02	1.60E-02
BIOCARTA	ALK pathway	9.49E-03	3.28E-02	3.12E-02	2.82E-03

### Heritability estimation

The previously identified 23 multiple myeloma risk SNPs were shown to account for 15.7% of the GWAS heritability, a relatively small fraction of the estimated 15.6% ( ± 4.7) net heritability explained by all common SNPs^5,7,18^. The identified interacting loci explain an additional 1.3% of the GWAS heritability in the UK cohort (1.5% in the German cohort) in the liability scale, which brings the collectively explained GWAS heritability to a modest 17% ( ± 2.4). However, as the heritability estimates are based on individual SNP associations and do not take into account the interaction term, the scope of interaction-identified heritability remains unanswered.

## Discussion

We have performed the first genome-wide interaction study on multiple myeloma to date. We discovered 16 unique multiple myeloma risk locus pairs. Several of the discovered SNPs depicted eQTL effects for nearby genes and they were implicated in the networks and pathways relevant for multiple myeloma biology. We also demonstrated that genes annotated to the loci are highly expressed in tissues and cells of the hemic-immune system.

Interferon regulatory factor 8 (*IRF8*) together with G protein subunit alpha Q (*GNAQ*) were discovered to be the paired risk loci with highest statistical significance. *IRF8* is reported to be a risk locus for immunoglobulin trait modulation, whereas GNAQ is a guanine nucleotide-binding protein that regulates B-cell development and survival^19,20^. IRF8 has many functions in regulating innate immunity and immune cell development, including B- and T-cells, dendritic cells, and myeloid cells^21^. In early development, IRF8 and IRF4 function redundantly to regulate transition of pre-B-cells to maturing B-cells. In the germinal center development, the roles of these IRFs are complementary: IRF8 directs early centroblast development, which is taken over by IRF4 as centrocytes mature into plasma cells. IRF8 induces activation-induced cytidine deaminase, which is a key enzyme catalyzing somatic hypermutations of plasma cells^21^. Similar to IRF4, IRF8 transcriptional activity in multiple myeloma may also be related to differentiation of T helper (T_H_) 17 cells, which have a regulatory effect on bone morphogenesis-related onset of multiple myeloma^22^. IRF8 has been reported to act as an intrinsic transcriptional inhibitor of T_H_17 cells, at least partly through its physical interaction with retinoic acid receptor-related orphan receptor RORγt^23^. As a confirmation of this signal, we identified enrichment of two circadian rhythm pathways regulated by two nuclear receptors, *RORA* and Rev-ErbA, which are crucial for the development of T_H_17 cells^24^. These findings together with our previous identification of rs4487645 at 7p15.3, as a modulator of IRF4 binding at an enhancer element of the c-Myc-interacting gene *CDCA7L* in multiple myeloma^25–27^, support the role of the genetic variants in IRF8 and its interacting partner in GNAQ in multiple myeloma susceptibility.

Another signaling cascade affecting immunoglobulin trait modulation, T_H_17 cell differentiation, and bone morphogenesis is the TGFβ pathway^28^, which was represented by three enriched pathways in our analysis. In multiple myeloma, enhanced bone resorption releases and activates TGFβ, which is a potent inhibitor of osteoblast differentiation and mineralization^29^. Our interaction analysis identified rs2834882 annotated to runt related transcription factor 1 (*RUNX1*) in interaction with rs2860107 at zinc finger CCHC-type containing 6 (*ZCCHC6*, alias *TUT7*). RUNX family transcriptional activities have been linked to TGFβ-induced IgA class switching, which is involved in multiple myeloma pathogenesis^19,30^. RUNX proteins also play a major role in cell differentiation, and RUNX1 is specifically regulating hematopoiesis^31^. Germline mutations in *RUNX1* cause familial platelet disorder with propensity to myeloid malignancies and somatic loss of RUNX1 function is related to several hematologic malignancies^29,32^. RUNX transcription factors are integral components of signaling pathways enforced by TGFβ family members including bone morphogenic proteins (BMPs). RUNX1 and RUNX2 are known modulators of BMP-2/7/9-induced osteoblast differentiation. *RUNX1* along with *RUNX2* is often found co-expressed in skeletal elements that regulate expression of BMP-2 and BMP-9.^33^. *RUNX2* regulatory activity in osteoblast differentiation is also regulated by transcription factor NKX3–2, whose expression was modulated by the sentinel SNP rs17362130 (Table 1)^16^. Additionally, ZCCHC11 and ZCCHC6 TUTase inhibitors are being investigated as potential agents for targeted therapy^34^.

Contextually in multiple myeloma, TGFβ induces differentiation arrest in osteoblasts, increases osteoclastogenesis, promotes angiogenesis, and suppresses host immunity in bone marrow microenvironment to create the so called multiple myeloma niche, thus enhancing multiple myeloma cell growth and survival^29^. TGFβ-activated transcription factors, SMADs also interact with chromatin binding proteins HDAC1 and HDAC2. *HDAC1* is a class I histone deacetylase gene and multiple myeloma patients with high protein levels of HDAC1 were shown to have poor progression-free and overall survival^35^. Moreover, inhibition of HDAC1 is demonstrated to induce multiple myeloma cell death^36^. We noted a significant interaction between a class II HDAC family member, *HDAC9* and neural cell adhesion molecule 2, *NCAM2*. Deregulation of *HDAC9* in cells of lymphoid lineage is believed to induce B-cell lymphoproliferative disorders including Waldenström macroglobulinemia and is associated with general poor prognosis in cancer^37,38^. *HDAC9* is also hypothesized to be responsible for lymphomagenesis by regulating growth and survival related pathways and by modulating of BCL6 and p53 tumor suppressor activity^38^. In germinal cells, it is shown to be co-expressed with BCL6, a therapeutic target for multiple myeloma^39^. Controlled cell cycle is critical for normal cellular growth, and its deregulation may possibly stimulate carcinogenesis.

HDACs are also shown to have role in transcriptional activity of NKX3–2, one of the eQTL targets of our study. It has been shown that BMP and SMAD signaling modulates the activity of NKX3–2 in a BMP-dependent manner by promoting NKX3–2 binding with SMAD1/4 and HDAC/SIN3A corepressor complex^40^. As HDAC inhibitors in general pose a vital role in cell cycle arrest induction and activation of intrinsic apoptotic mechanism, our observation leads to speculation that a common variation in 7p21.1 may predispose to multiple myeloma progression.

The recent meta-analyses have pointed out apoptosis and autophagy, B-cell and plasma cell development, cell cycle regulation and genomic stability, chromatin remodeling and immunoglobulin production as the main pathways deregulated by the identified multiple myeloma susceptibility loci^5,7^. We identified causally related genes implicated in apoptosis, such as *RIPK1* and *SERPINB9*. Among the interacting loci we identified genes involved in B-cell development and immunoglobulin production, such as *GNAQ* and *IRF8* and the TGFβ pathway and genes modifying the chromatin state, such as *HDAC9*. As TGFβ signaling is modified by ubiquitination and deubiquitination^41^, our study also support the importance of ubiquitin-proteasome signaling in multiple myeloma, which was highlighted by the meta-analysis together with the mechanistic target of rapamycin (mTOR) signaling as targets for already approved drugs in multiple myeloma^7^.

In conclusion, our findings provide further evidence that multiple myeloma is a genetically heterogeneous disease with inherited genetic susceptibility loci contributing excess risk via regulation of an assortment of regulatory networks and pathways. The two major signaling cascades we identified, TGFβ signaling through its signal transducers SMADs and circadian rhythm regulation by *RORA* and Rev-ErbA, integrate immunoglobulin trait modulation, T_H_17 cell differentiation, and bone morphogenesis, and may provide a mechanistic link between the predisposition markers and intrinsic multiple myeloma biology.

## Methods

### Ethics

Patient samples and relevant clinico-pathological information was obtained conditional on written informed consent and was subject to approval of corresponding ethical review boards at respective study centers in accordance with the tenets of Declaration of Helsinki including Myeloma-IX trial by the Medical Research Council (MRC) Leukemia Data Monitoring and Ethics committee (MREC 02/8/95, ISRCTN68454111), the Myeloma-XI trial by the Oxfordshire Research Ethics Committee (MREC 17/09/09, ISRCTN49407852) and Ethical Commission of medical faculty, University of Heidelberg (229/2003, S-337/2009, AFmu-119/2010).

### Genome-wide association studies

Diagnosis of multiple myeloma (ICD-10 C90.0) adhered to the guidelines established by World Health Organization. Samples retrieved from all subjects were either before treatment or at presentation.

The UK GWAS^5^ consisted of 2282 cases (1755 male (post quality control (QC)) recruited through the UK MRC Myeloma-IX and Myeloma-XI trials (ISRCTN68454111: Myeloma- X http://www.isrctn.com/search?q=ISRCTN68454111 and ISRCTN49407852: Myeloma- XI http://www.isrctn.com/search?q=ISRCTN49407852). DNA was extracted from EDTA-venous blood samples (90% before chemotherapy) and genotyped using Illumina Human OmniExpress-12 v1.0 arrays (Illumina). Controls were recruited from publicly accessible data generated by the Welcome Trust Case Control Consortium (WTCCC) from the 1958 Birth Cohort (58C; also known as the National Child Development Study) and National Blood Service. The control population comprised of 5197 individuals (2628 male (post QC)). Genotyping of these controls was conducted using Illumina Human 1–2 M-Duo Custom_v1 Array chips (www.wtccc.org.uk).

The German GWAS^5^ comprised 1717 cases (981 male (post QC); mean age at diagnosis: 59 years). The cases were ascertained by the German-Speaking Multiple Myeloma Multicenter Study Group (GMMG) coordinated by the University Clinic, Heidelberg (ISRCTN06413384: GMMG-HD3 http://www.isrctn.com/search?q=ISRCTN06413384; ISRCTN64455289: GMMG-HD4 http://www.isrctn.com/search?q=ISRCTN64455289; and ISRCTN05745813: GMMG-MM5 http://www.isrctn.com/search?q=ISRCTN05745813). DNA was prepared from EDTA-venous blood or CD-138-negative bone marrow cells ( < 1% tumor contamination). Genotyping of these samples was performed using Illumina Human OmniExpress-12 v1.0 arrays (Illumina). For controls, we used genotype data on 2107 healthy individuals, enrolled into the Heinz Nixdorf Recall (HNR) study genotyped using either Illumina HumanOmni1-Quad_v1 or OmniExpress-12 v1.0 arrays. Out of the whole recruited control population, 2069 (1028 male) remained after QC.

### Analysis of GWAS

Quality control of the GWAS data was performed according to predetermined benchmarks already published^5^. In summary, inclusion of samples was initially liable to successful genotyping of $\ge 95\%$ of the SNPs. Duplicates, first-degree relatives, and closely related individuals were removed with an identity-based-test (IBS) score$\ge 0.80$. Genetic heterogeneity was assessed with principal component analysis using dissimilarity measure calculated with our SNP panel and genome-wide IBS distances in reference to the HapMap samples. In each of the samples, SNPs having a minor allele frequency $< 0.01$ and call rate of $< 95\%$ were filtered out. SNPs were also excluded subject to departure from Hardy–Weinberg equilibrium at $P < 1 \times 10^{ - 5}$ in controls.

### Genome-wide interaction study

Analyses were primarily undertaken using PLINK (v1.09), CASSI (v3), METAINTER, and R (v3.4.0) software. The interaction between each SNP pair and the risk of multiple myeloma was assessed with Pearsonian product moment correlation coefficient-based test inspired by Wellek-Ziegler statistics given by the formula^42^:

$$T_{{{{\rm{WZ}}}_{{\mathrm{case}}/{\mathrm{control}}}}} = \frac{{({r}_{A} - {r}_{N})^2}}{{{\mathrm{Var}}\left( {r}_{A} \right) + {\mathrm{Var}}({r}_{N})}}$$

Where *r* is pearsonian correlation coefficient statistics defined by Wellek and Ziegler^43^. *r*_*A*_ and *r*_*N*_, respectively, represent the statistics calculated among cases and controls separately. To this end, we used CASSI software. Genomic resolution of the whole interaction test space was deflated with default predefined control option where all the variants having weaker signal (single marker association$P > 1.0 \times 10^{ - 3}$) were excluded^10^. We performed the interaction test in the German and UK cohorts separately and meta-analyzed the results to strengthen the signals from co-occurring interacting pairs. To conduct meta-analysis METAINTER software was employed assuming a fixed effects model. Gamma approximated negative sum of log transformed interaction statistics from each of the two sets were considered as the test statistic for each variant pair and was tested with a weighted Chi-square statistic with four degrees of freedom^44^. Odds ratio and associated 95% confidence intervals were calculated with unconditional logistic regression with independence assumption among each component of SNP pairs.

### Expression quantitative trait loci analysis

Investigation of true regulatory effects of the SNPs identified with the interaction study was undertaken by analyzing eQTL data obtained from malignant plasma cells of 665 multiple myeloma patients (389 male) enrolled in the German multiple myeloma trials conducted in Heidelberg University clinic. CD-138 purified plasma cells were used for gene expression profiling using Affymetrix U133 2.0 plus arrays. The expression data was submitted to Gene Expression Omnibus (E-MTAB-2299). All analyses were undertaken with R software. GC-RMA was used to normalize the expression data and genes with transformed log2 expression < 3.5 in at least 95% of the samples were excluded from further consideration. With exclusive consideration of autosomal genes, 9722 genes remained after QC. We investigated the correlative relationship between the identified individual risk SNPs within 1 Mb window (cis-eQTL analysis), which narrowed the candidates to a set of 239 genes. A Holm-Sidák corrected level of significance for discovery was determined at $< 0.0002$ i.e., $\left[ {1 - (1 - 0.05)^{1/239}} \right]$ on 239 probes corresponding to all the variants. Robust regression on a transformed Huber function was employed to model the qualitative traits as it warrants higher detection power in moderately contaminated sample^45^. To avoid singularity of the argument space, variants in high linkage disequilibrium were discarded from consideration.

To extend the investigation of relation between SNP genotype and expression levels of genes and to identify causal candidates rather than mere associative pairings, we adapted SMR analysis as per Zhu et al.^12^. In summary, if we nominate effect size of a differentially expressed gene *X* on coherence of a phenotype *Y* to be $\beta _{XY}$ and consider the SNP genotypes to be the instrumental variable actively regulating both gene expression and the phenotype, then we can linearly estimate $\beta _{XY}$ by comparative effect-sizes.

$$\hat \beta _{XY} = \frac{{\hat \beta _{ZY}}}{{\hat \beta _{ZX}}}$$

Where $\hat \beta _{ZY}$is the estimated effect size of genetic factor on the phenotype, which is assessed as GWAS effect size and $\hat \beta _{ZX}$is that of the genetic factor of the expression levels of the genes, i.e., the eQTL effect size. We need not distinguish pleiotropic effect from high linkage co-occurrence since the SNPs in linkage disequilibrium demonstrated equal effect size. Thus, reliability of causal genes was tested with the approximated SMR statistic against $\chi _1^2$.

### Network enrichment

A protein–protein interaction confidence network was formulated with STRING (v10.5, 04/18/2018). Interactions between two proteins were calculated based on the likelihood confidence of an edge between the two nodes and was transposed to a scale of 0 to 1 (1 representing high confidence). We built our network with the genes annotated by the interaction-discovered SNPs and eQTL analysis; in addition to that, first batch of first-degree predicted interactive nodes were included given a confidence score > 0.99. Erroneous discovery was restricted at 10%, which rendered a protein–protein interaction network index *P* < 0.0054 (observed number of interactions were tested against expected number of interactions with chi-square statistic with one degree of freedom).

### Pathway enrichment

Initial in silico pathway enrichment was performed with the PASCAL tool interrogating the GWAS obtained summary statistics^46^. To create mapping of genes and single entity gene-fusions with PASCAL, we considered all genes within 20 kb upstream and downstream to an index SNP and fused all the corresponding/flanking genes together when the genes were found affecting same pathway(s). Sum of chi-square statistics with individual one degree of freedom was computed by summing over association statistics corresponding to each pathway. Enrichment scores of individual pathways were subsequently obtained by a test assuming chi-square distribution with degrees of freedom equal to the cardinality of fused gene sets.

### Tissue and cell type enrichment

DEPICT was employed to analyze tissue and cell type enrichment that predicts differential regulation of the selected loci on any of the Medical Subject Heading (MeSH) annotations^47^. To this end, 209 such annotations were tested for 37,427 inbuilt backend human microarrays on the Affymetrix HGU133a2.0 array platform. The tissue/cell type enrichment is thus performed on the normalized expression matrix after subjecting it to user selected dimension reduction criteria. SNP pairs discovered with interaction test represented 12 unique mapped regions against which the enrichment was tested, hence we tested against a conservative threshold of significance at negative log transformed *P*-value of 2.37 correcting for multiple testing, which retains the false discovery rate at < 5%^48^.

### Heritability analysis

As hypothesized by earlier studies, heritability estimates of complex diseases with polygenic origin are more robust with lifetime risk compared to population prevalence^49^. Following this notion, lifetime risk of multiple myeloma was assumed (0.007 for UK and 0.006 for German population; https://www.cancerresearchuk.org/health-professional/cancer-statistics/statistics-by-cancer-type/myeloma; https://www.krebsdaten.de/Krebs/EN/Home/homepage_node.html) to ascertain heritability of multiple myeloma explained by the risk SNPs discovered in the two different cohorts separately. Principal components were included to adjust for inflation as covariates. Genome-wide Complex Trait Analysis was used to estimate the genetic variance ascribable to the identified loci at a liability scale^50,51^.

### Reporting summary

Further information on experimental design is available in the Nature Research Reporting Summary linked to this article.

## Supplementary information

Supplementary Data 1

Supplementary Data 2

Supplementary Data 3

Description of Additional Supplementary Files

Reporting Summary

## Data Availability

SNP genotyping data that support the findings of this study have been deposited in Gene Expression Omnibus with accession codes GSE21349, GSE19784, and GSE15695; in the European Genome-phenome Archive (EGA) with accession code EGAS00000000001; and in the database of Genotypes and Phenotypes (dbGaP) with accession code phs000207.v1.p1. Expression data that support the findings of this study have been deposited in EMBL-EBI with accession code E-MTAB-2299. The remaining data are contained within the paper and Supplementary Data or are available from the author upon request.
