# Supplementary material for: Genome-wide interaction and pathway-based identification of key regulators in multiple myeloma
Source: Commun Biol. 2019 Mar 4;2:89. doi: 10.1038/s42003-019-0329-2 (PMC6399257; doi:10.1038/s42003-019-0329-2)
Supplement: Supplementary file 4 — Description of Additional Supplementary Files [file 42003_2019_329_MOESM4_ESM.docx]

**Description of Supplementary Data**

Supplementary Data 1: Genome-wide interaction analysis of the UK and the German MM samples and their meta-analysis.

Supplementary Data 2. Tissue and cell enrichment analysis with DEPICT

Supplementary Data 3. Pathway analysis with PASCAL (Including summary statistics and meta-data for all sets of iterations)
