# Supplementary material for: Genome-wide interaction and pathway-based identification of key regulators in multiple myeloma
Source: Commun Biol. 2019 Mar 4;2:89. doi: 10.1038/s42003-019-0329-2 (PMC6399257; doi:10.1038/s42003-019-0329-2)
Supplement: Supplementary file 5 — Reporting Summary [file 42003_2019_329_MOESM5_ESM.pdf]

natureresearch

Corresponding author(s): Asta Försti

Reporting Summary

Nature Research wishes to improve the reproducibility of the work that we publish. This form provides structure for consistency and transparency in reporting. For further information on Nature Research policies, see Authors & Referees and the Editorial Policy Checklist.

Statistical parameters

When statistical analyses are reported, confirm that the following items are present in the relevant location (e.g. figure legend, table legend, main text, or Methods section).

n/a	Confirmed	
☐	☒	The exact sample size (*n*) for each experimental group/condition, given as a discrete number and unit of measurement
☐	☒	An indication of whether measurements were taken from distinct samples or whether the same sample was measured repeatedly
☐	☒	The statistical test(s) used AND whether they are one- or two-sided<br>*Only common tests should be described solely by name; describe more complex techniques in the Methods section.*
☐	☒	A description of all covariates tested
☐	☒	A description of any assumptions or corrections, such as tests of normality and adjustment for multiple comparisons
☐	☒	A full description of the statistics including central tendency (e.g. means) or other basic estimates (e.g. regression coefficient) AND variation (e.g. standard deviation) or associated estimates of uncertainty (e.g. confidence intervals)
☐	☒	For null hypothesis testing, the test statistic (e.g. *F*, *t*, *r*) with confidence intervals, effect sizes, degrees of freedom and *P* value noted<br>*Give P values as exact values whenever suitable.*
☒	☐	For Bayesian analysis, information on the choice of priors and Markov chain Monte Carlo settings
☒	☐	For hierarchical and complex designs, identification of the appropriate level for tests and full reporting of outcomes
☒	☐	Estimates of effect sizes (e.g. Cohen's *d*, Pearson's *r*), indicating how they were calculated
☒	☐	Clearly defined error bars<br>*State explicitly what error bars represent (e.g. SD, SE, CI)*

Our web collection on statistics for biologists may be useful.

Software and code

Policy information about availability of computer code

Data collection	No software tool is involved in data collection
Data analysis	Unix platform based tools: PLINK, SNPTEST, IMPUT2, QCTOOL, CASSI, INTERSNP, GCTA, METAINTER, PASCAL, DEPICT<br>Analysis platform: R, Python, Java<br>No custom code is in use

For manuscripts utilizing custom algorithms or software that are central to the research but not yet described in published literature, software must be made available to editors/reviewers upon request. We strongly encourage code deposition in a community repository (e.g. GitHub). See the Nature Research guidelines for submitting code & software for further information.

Data

Policy information about availability of data

All manuscripts must include a data availability statement. This statement should provide the following information, where applicable:

- Accession codes, unique identifiers, or web links for publicly available datasets
- A list of figures that have associated raw data
- A description of any restrictions on data availability

All data used are already deposited to publicly accessible domains and accession numbers and reference links are provided wherever applicable

Field-specific reporting

Please select the best fit for your research. If you are not sure, read the appropriate sections before making your selection.

☒ Life sciences ☐ Behavioural & social sciences ☐ Ecological, evolutionary & environmental sciences

For a reference copy of the document with all sections, see nature.com/authors/policies/ReportingSummary-flat.pdf

Life sciences study design

All studies must disclose on these points even when the disclosure is negative.

Sample size	Case-control set up: 3999 cases and 7266 controls
Data exclusions	Sample/variant exclusion is performed according to standard quality control protocol for phasing and imputation in GWAS. Additionally no subjective sampling was in effect.
Replication	Validation of results is ensured via different in-silico approaches, but no immediate replication is discussed
Randomization	Not relevant in this observational set up
Blinding	Not relevant in this observational set up

Reporting for specific materials, systems and methods

Materials & experimental systems

n/a	Involved in the study
☒	☐ Unique biological materials
☒	☐ Antibodies
☒	☐ Eukaryotic cell lines
☒	☐ Palaeontology
☒	☐ Animals and other organisms
☐	☒ Human research participants

Methods

n/a	Involved in the study
☒	☐ ChIP-seq
☒	☐ Flow cytometry
☒	☐ MRI-based neuroimaging

Human research participants

Policy information about studies involving human research participants

Population characteristics

Diagnosis of MM (ICD-10 C90.0) adhered to the guidelines established by World Health Organization. Samples retrieved from all subjects were either before treatment or at presentation.
The UK GWAS5 consisted of 2282 cases (1755 male (post quality control (QC))) recruited through the UK MRC Myeloma-IX and Myeloma-XI trials (ISRCTN68454111: Myeloma- X http://www.isrctn.com/search?q=ISRCTN68454111 and ISRCTN49407852: Myeloma- XI http://www.isrctn.com/search?q=ISRCTN49407852). DNA was extracted from EDTA-venous blood samples (90% before chemotherapy) and genotyped using Illumina Human OmniExpress-12 v1.0 arrays (Illumina). Controls were recruited from publicly accessible data generated by the Welcome Trust Case Control Consortium (WTCCC) from the 1958 Birth Cohort (58C; also known as the National Child Development Study) and National Blood Service. The control population is comprised of 5197 individuals (2628 male (post QC)). Genotyping of these controls was conducted using Illumina Human 1-2M-Duo Custon_v1 Array chips (www.wtccc.org.uk).

The German GWAS5 comprised 1717 cases (981 male (post QC); mean age at diagnosis: 59 years). The cases were ascertained by the German-Speaking Multiple Myeloma Multicenter Study Group (GMMG) coordinated by the University Clinic, Heidelberg (ISRCTN06413384: GMMG-HD3 http://www.isrctn.com/search?q=ISRCTN06413384; ISRCTN64455289: GMMG-HD4 http://www.isrctn.com/search?q=ISRCTN64455289; and ISRCTN05745813: GMMG-HD5 http://www.isrctn.com/search?q=ISRCTN05745813). DNA was prepared from EDTA-venous blood or CD138-negative bone marrow cells (<1% tumor contamination). Genotyping of these samples was performed using Illumina Human OmniExpress-12 v1.0 arrays (Illumina). For controls, we used genotype data on 2,107 healthy individuals, enrolled into the Heinz Nixdorf Recall (HNR) study genotyped using either Illumina HumanOmni1-Quad_v1 or 1428 OmniExpress-12 v1.0 arrays. Out of the whole recruited control population, 2069 (1028 male) remained post QC.

Recruitment

Trial recruited patients: See above
